# Critical Appraisal of the MTT Assay in the Presence of Rottlerin and Uncouplers

**DOI:** 10.1007/s12575-009-9020-1

**Published:** 2009-12-03

**Authors:** Emanuela Maioli, Claudia Torricelli, Vittoria Fortino, Filippo Carlucci, Valentina Tommassini, Adriana Pacini

**Affiliations:** 1 Department of Physiology, University of Siena, via Aldo Moro, 7-53100, Siena, Italy; 2 Department of Internal Medicine, Endocrine-Metabolic Science and Biochemistry, University of Siena, via Aldo Moro, 7-53100, Siena, Italy

**Keywords:** Rottlerin, MCF-7 cell, MTT, LDH, FCCP, mitochondrial uncoupling

## Abstract

Rottlerin is a natural product isolated from *Mallotus philippinensis*. This polyphenolic compound, originally described as a selective inhibitor of PKCδ, can inhibit many other PKC-unrelated kinases and has a number of biological actions, including mitochondrial uncoupling effects. We recently found that Rottlerin inhibits the transcription factor nuclear factor κB in different cell types, causing downregulation of cyclin D1 and growth arrest. The present study was carried out to clarify the surprising lack of effect of Rottlerin on MCF-7 cell viability, assessed by the 3-(4,5-dimethylthiazol-2-yl)-2,5-diphenyl tetrazolium bromide (MTT) test. We found that Rottlerin causes overestimation of the MTT test, leading to inconsistent results between cell number and cell viability. Rottlerin, however, strongly differs from other antioxidant polyphenols, which directly reduce tetrazolium salts, since it does not exhibit any reactivity toward the tetrazolium salts in vitro nor does it modulate lactate dehydrogenase activity. The interference in the MTT assay occurred only in cultured cells, concomitantly with a decrease in the energy charge. Because the same MTT overestimation was observed in the presence of uncoupling agents, we conclude that the Rottlerin artifact is linked to its uncoupling action that, by accelerating oxidative chain, accidentally results in enhanced MTT reduction. These results suggest caution in the use of the MTT assay in the presence of Rottlerin and uncouplers in general.

## 1. Introduction

**Figure 1:**
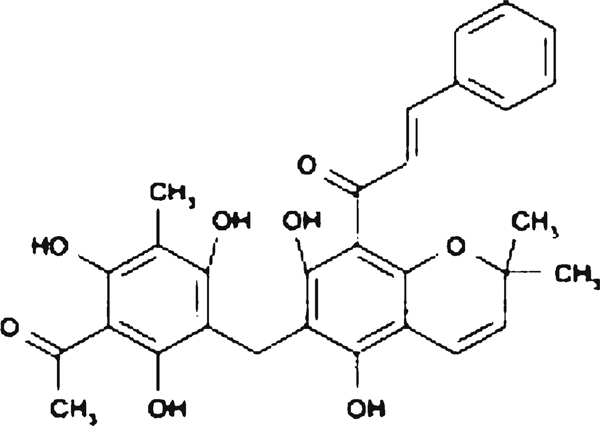
**Rottlerin structure**.

Rottlerin (also called mallotoxin or kamala), is a 5,7-dihydroxy-2,2-dimethyl-6-(2,4,6-trihydroxy-3-methyl-5-acetylbenzyl)-8-cinnamoyl-l,2- chromene, a pigmented plant product isolated from *Mallotus philippinensis* (Figure 1). Since 1994, Rottlerin has been used as a PKCδ inhibitor [1] although the selectivity of Rottlerin in inhibiting the PKCδ isoform has been recently questioned [2,3] and ascribed to a likely indirect effect mediated by mitochondrial uncoupling and decrease in ATP content [4]. Our laboratory showed for the first time that Rottlerin can prevent, independently from PKC, the activation of the transcription factor nuclear factor κB (NFκB), induced by either phorbol ester or oxidative stress in MCF-7 cells [5], HaCaT keratinocytes [6] and HT-29 cells (unpublished results), whose growth resulted to be arrested because of downregulation of cyclin D1, at both the protein and mRNA levels. Although the molecular mechanism is not definitively clarified, the prevention of the NFκB activation process was likely achieved through both Rottlerin inhibition of protein kinases [7,8] and Rottlerin free radicals scavenging activity [9]. Indeed, NFκB can be activated by a number of pathways and is a redox-sensitive transcription factor for key molecules involved in inflammation, cancer progression, cell cycle control, and protection against apoptosis [10].

However, in our previous paper [5], we found that MCF-7 cell viability was not altered by a 24-h Rottlerin treatment, a result that was in evident conflict with the inhibition of NFkB and cell proliferation, as evaluated by [^3^H]-thymidine incorporation into DNA. Since the measurement of cell viability was based on the reduction of 3-(4,5-dimethylthiazol-2-yl)-2,5-diphenyl tetrazolium bromide (MTT) to formazan crystal by mitochondrial dehydrogenases [11], in the current study we revisited our previous results, checking for a possible interference of Rottlerin in the MTT assay.

## 2. Materials and methods

### Materials

All chemicals and materials for cell culture (unless otherwise indicated) were obtained from Sigma (Milan, Italy). Lactate dehydrogenase (LDH) assay kit was purchased from Sclavo Diagnostics (Siena, Italy). Rottlerin was dissolved in dimethyl sulfoxide (DMSO). Carbonylcyanide-4-(trifluoromethoxy)-phenylhydrazone (FCCP) was dissolved in 95% ethanol.

### Cells and culture conditions

MCF-7 cells, purchased by Istituto Zooprofilattico Sperimentale della Lombardia e dell'Emilia-Romagna, Brescia, Italy, were grown in a humidified atmosphere (95% air/5% CO_2_) at 37°C in MEM containing 10% FBS, Na-pyruvate (1 mM), and antibiotics.

Primary human microvascular endothelial cells (HMVEC), purchased by LONZA (Milano, Italy), were cultured in EBM-2 medium supplemented with EGM-2 Single Quots (LONZA), and experiments were performed on cultures from passages 3 to 9.

After reaching subconfluence, cells were incubated in serum-free medium for 24 h and then subjected to treatments in 2.5% serum.

### Cell counting

The number of cells, cultured in 25-cm^2^ culture flasks (Falcon, Perugia, Italy) was evaluated by detaching with trypsin solution (0.05% trypsin–0.02% sodium EDTA) and counting using a Bürker chamber and trypan blue solution (0.2% *w*/*v* final dye concentration). Viable MCF-7 cell number counts were obtained at 1-, 2-, 4-, 15-, and 24-h incubation in the presence or absence of Rottlerin 5 and 20 μM.

Viable HMVEC cell number counts were obtained at 24-h incubation in the presence or absence of 20 μM Rottlerin.

Three replicate counts were determined by the same operator at each time point. The data were presented as proportional viability (%) by comparing the Rottlerin-treated with the vehicle-treated cells, whose viability was assumed to be 100%.

### MTT assay

Cell viability was assessed using the MTT colorimetric assay. MTT is taken up into cells by endocytosis or protein-facilitated mechanism and reduced, mainly by mitochondrial enzymes, to yield a purple formazan product which is largely impermeable to cell membranes, thus resulting in its accumulation within living cells. Solubilization of the cells results in the liberation of the purple product which can be detected using a colorimetric measurement. The ability of cells to reduce MTT provides an indication of the mitochondrial integrity and activity which, in turn, may be interpreted as a measure of cell number/proliferation/viability/survival/toxicity.

Operatively, 100 μl of cell suspension was inoculated to each well of 96-well plates at the density of 2 × 10^4^ cells/well (the area of each well was 0.32 cm^2^). After 24 h of culture, the medium was removed by aspiration and replaced with 100 μl of experimental medium. The treatments were performed with 5 and 20 μM Rottlerin for 1, 2, 4, 15, and 24 h. After incubation, cells were observed under a contrast phase microscope before adding MTT solution, prepared fresh as 5 mg/ml in H_2_O, filtered through a 0.22-μm filter, and kept for 5 min at 37°C. MTT solution (10 μl) was added to each well, and the plates were incubated in the dark for 4 h at 37°C.

To check for the direct effect of Rottlerin on the formazan production, a parallel set of experiments was carried out in cell-free plates. The followed procedure was the same as described above with some modifications: (a) The Rottlerin doses were 5, 20, 50, and 100 μM; (b) the incubation time with MTT was prolonged from 4 up to 24 h; (c) different media (DMEM, MEM, and RPMI 1640), enriched or not with 10% serum or 20 μM NAD or NADH, were tested.

In another set of experiments MCF-7 cells were treated for 30 min with equal doses (5 and 20 μM) of Rottlerin or the chemical uncoupler FCCP, before incubation with 10 μl of MTT for 1 h at 37°C.

HMVEC were treated for 24 h with 20 μM Rottlerin before incubation with MTT for 4 h.

At the end of each experiment, the medium was removed by inverting and tapping the plates and 100 μl solution of 4% HCl 1 N in isopropanol was added to immediately dissolve the formazan crystals. Absorbance at 570 nm was read on a Multiwell scanning spectrophotometer (Sclavo, Siena, Italy), and the results were expressed as a percentage (%) of the control (vehicle alone).

The medium from the plates without cells was collected and both read directly and centrifuged before solubilization of (eventual invisible) formazan crystals. In this experiment, the reducing agent dithiothreitol was used as a positive control.

### LDH assay

LDH assay was performed in culture medium of untreated confluent cells by using a commercial kit (Sclavo Diagnostics, Siena) based on the transformation of pyruvate to lactate by LDH, at pH 7.5, in the presence of NADH coenzyme. The transformation of NADH to NAD+ is accompanied by a decrease in absorbance (A) at 340 nm, which correlates with the LDH activity. The change of absorbance, in the absence or presence of different doses of Rottlerin, was recorded over a 0.5- to 4.5-min period, and the relative Δ*A*/min was calculated. The change in absorbance was converted to LDH international units per liter (U/l) by the following calculations: Δ*A*/min ⋅ (tV ⋅ 1,000/EMC ⋅ *l* ⋅ sV), where tV is the total volume, EMC is the NADH extinction micromolar coefficient (6.22 cm^2^ μmol at 340 nm), *l* is the light pathlength (1 cm), and sV is the sample volume.

### Evaluation of nucleotide content

Nucleotides were evaluated as previously described [12]. The cells (5 × 10^6^) were homogenized in 2.7 N perchloric acid in 0.5-ml tubes using a Sigma nylon motor pestle. Extracts were then centrifuged (12,000×*g* for 10 min) in a cooled microfuge. The supernatant was neutralized with 2.7 N KOH; potassium perchlorate was removed by a subsequent centrifugation at 12,000×*g* for 3 min. Aliquots of the extracts were analyzed by capillary zone electrophoresis (CZE), as afterward reported. The method permits to determine high-energy phosphates (ATP, ADP, and AMP) from which it is possible to calculate the energy charge of adenylates.

### Capillary zone electrophoresis

For electrophoretic separations a Beckman Coulter P/ACE MDQ instrument (Beckman Coulter, Fullerton, CA, USA) was used. Analysis were performed in a Beckman Coulter eCAP™ uncoated fused-silica capillary (65.0 cm × 75 μm i.d.), with the window at a distance of 55.0 cm. The results were read over the range 190–300 nm and analyzed at 254 nm. Between runs capillary was washed with 0.1 mol/l NaOH for 30 s and running buffer for 60 s. The background electrolyte was borate buffer (20 mM), containing SDS (30 mM). The conditions were pH 10.00, 20 kV, and 1.0 psi for 5 s of pressure injection at 25°C, for sample and standard solutions. The electric field was 306 V/cm with a current of approximately 120 μA.

### Statistical analysis

The significant difference between control and treated cells was statistically analyzed by paired Student's *t* test (*p* < 0.05).

## 3. Results

### 3.1. Rottlerin interference in the MTT assay

**Figure 2:**
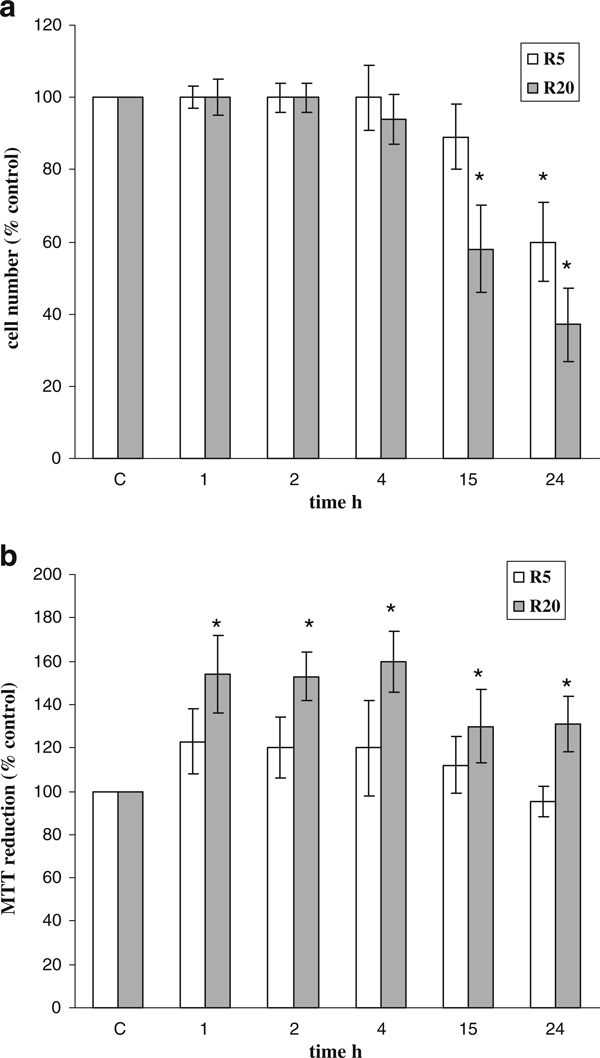
**Effect of Rottlerin on cell number and viability assessed by direct cell counting and MTT assay, respectively**. MCF-7 cells were treated with 5 and 20 μM Rottlerin (*R5* and *R20*) before adding MTT. **a** Between 1 and 24 h later, cell number was determined, and **b** cell viability was measured as described in "Materials and methods". Results are expressed as % of the control (100%). Values are the average of three separate experiments in quadruplicate and are expressed as mean ± SD. *p < 0.05.

Consistently with the decrease in [^3^H]-thymidine incorporation into DNA previously observed [5], 5 and 20 μM Rottlerin caused a time and a dose-dependent decrease in cell number, although the dose of 5 μM did not cause statistically significant changes before 24 h (Figure 2a). However, as shown in Figure 2b, the MTT colorimetric assay strongly underestimated the growth inhibitory activity of 20 μM Rottlerin and absolutely reversed the result, suggesting a direct Rottlerin action on the tetrazolium salt. Rottlerin (5 μM) exhibited the same trend of 20 μM although the results were not statistically significant at any time point. When the intensity of the reduced product color at 570 nm was normalized to cell number (absorbance/cell number), the stimulation of MTT reduction by Rottlerin was evident (Table 1). Rottlerin, however, did not reduce MTT in vitro at doses up to 100 μM and for prolonged times (24 h), in several incubation conditions, i.e., in different culture media and in the presence or absence of serum, NAD or NADH (not shown).

**Table 1:** MTT reduction normalized to cell number

	MTT/cell number	
	
Time (h)	R5	R20
1	1.23	1.54
2	1.20	1.53
4	1.20	1.70
15	1.25	2.24
24	1.58	3.49

The MTT (% control)/cell number (% control) ratio was calculated in the samples treated with 5 and 20 μM Rottlerin (R5 and R20, respectively) at the indicated time points

**Figure 3:**
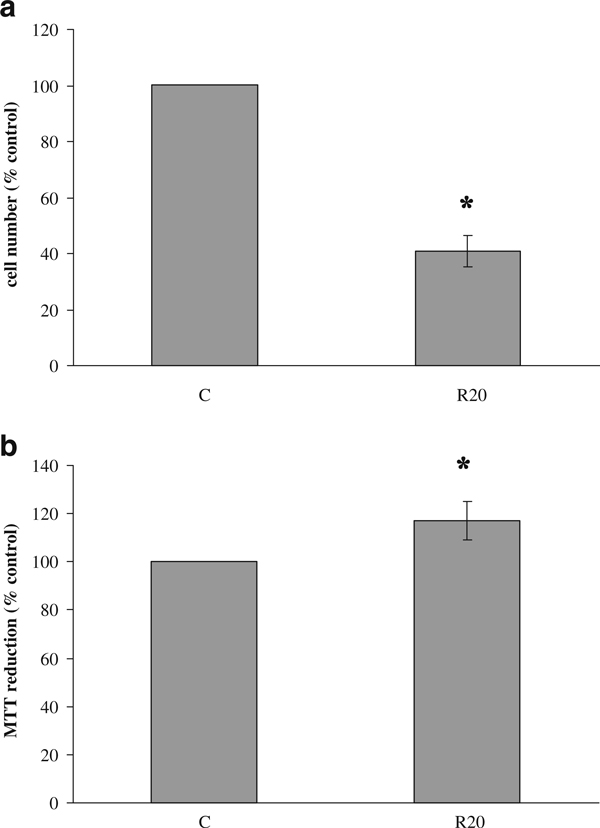
**Effect of Rottlerin on HMVEC cell number and viability assessed by direct cell counting and MTT assay, respectively**. HMVEC were treated with 20 μM Rottlerin (*R20*) before adding MTT. **a** Twenty-four hours later, cell number was determined, and **b** cell viability was measured as described in "Materials and methods". Results are expressed as % of the control (100%). Values are the average of three separate experiments in quadruplicate and are expressed as mean ± SD. *p < 0.05.

As shown in Figure 3, an overestimation of the MTT assay with respect to cell number was also observed in HMVEC, after a 24-h treatment with 20 μM Rottlerin.

### 3.2. LDH assay in the presence of Rottlerin

As shown in Table 2, the kinetic of NADH oxidation by LDH is unchanged in the presence of 5 (not shown) and 20 μM Rottlerin with respect to the control, as it results from the very similar Δ*A* at the different times, although the absolute optical densities (OD; unchanged in the presence of 5 μM Rottlerin) are higher in the presence of 20 μM Rottlerin due to its intrinsic absorbance at 340 nm. Anyway, LDH activity and quantification are not altered by Rottlerin, the spectrophotometric interference of which does not invalidate LDH quantification.

**Table 2:** LDH absorbance changes at 340 nm as a function of time in the presence or absence of Rottlerin

	DMSO		20 μM Rottlerin	
		
Time	OD	Δ*A*	OD	Δ*A*
30 s	1.248		1.365	
1.5 min	1.211	0.037	1.320	0.045
2.5 min	1.180	0.031	1.296	0.024
3.5 min	1.147	0.033	1.262	0.034
4.5 min	1.122	0.025	1.222	0.040
Mean Δ*A*/min	0.031		0.035	
LDH (U/l)	503		568	

The values reported in the table are representative of two LDH assays in the same sample and conditions

### 3.3. Rottlerin effect on the energy charge

**Figure 4:**
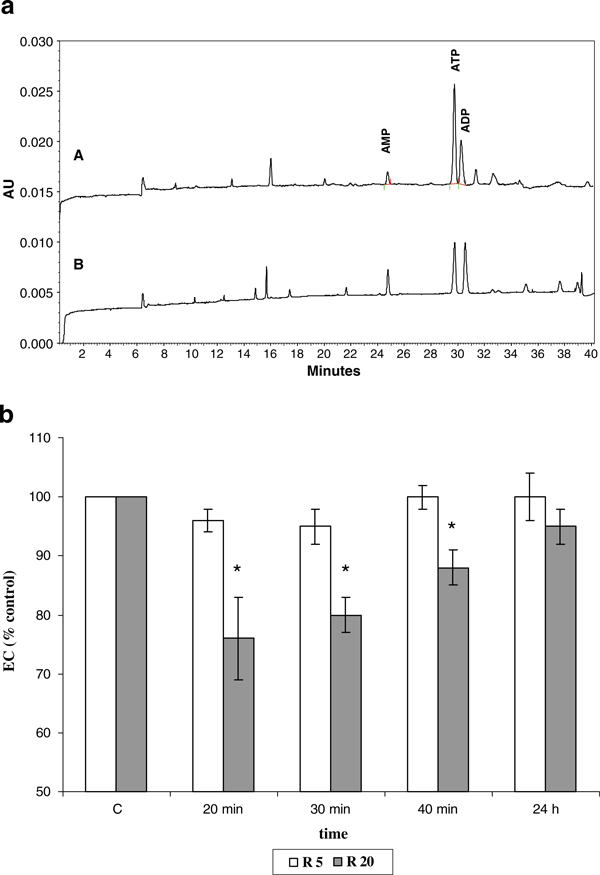
**Effect of Rottlerin on the energy charge (*EC*)**. Cells were treated with 5 and 20 μM Rottlerin (*R5* and *R20*), and high-energy phosphates (*ATP*, *ADP*, and *AMP*) were determined by CZE, as described in "Materials and methods". **a** Representative electropherogram of perchloric acid extracts from MCF-7 cells. *A* Control sample, *B* 30-min incubation with 20 μM Rottlerin. *AU* absorbance units. **b** The EC, represented by the ATP + ADP + AMP/0.5(ATP + AMP) ratio, was calculated at the indicated time points and expressed as % of the control (100%). Values are the average of three separate experiments and are expressed as mean ± SD. *p < 0.05.

Because an immediate sign of mitochondrial uncoupling is the drop in cell phosphorylation potential, we evaluated the Rottlerin uncoupling effect by detecting the levels of all components of the adenylate pool using CZE (Figure 4a) and then calculating the energy charge, represented by the ATP + ADP + AMP/0.5(ATP + AMP). Rottlerin uncoupling effect is clearly evident at the dose of 20 μM, appearing as a fall in the cellular energy charge (Figure 4b). The capacity to form high-energy phosphates was maximally compromised after 20 min (24% decrease) and was recovered slowly thereafter. At 24 h, cells recovered about 95% of the basal energy status, likely by compensatory metabolism. Rottlerin, at the dose of 5 μM, exerted a less evident effect (not statistically significant) on the cellular energy status, which was weakly compromised after 20–30 min treatment (4–5% decrease) and was completely recovered after 40 min.

### 3.4. Effect of mitochondrial uncoupling on MTT reduction

**Figure 5:**
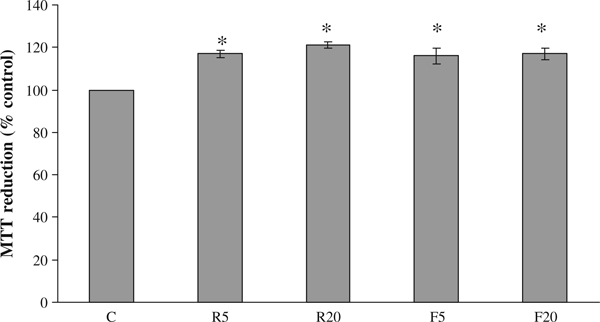
**Effect of Rottlerin and FCCP on MTT assay**. Cells were treated for 30 min with 5 and 20 μM Rottlerin and FCCP (*R5*, *R20* and *F5*, *F20*, respectively) or vehicle alone (DMSO and ethanol, respectively) and MTT reduction was evaluated as described in "Materials and methods". Values are the average of three separate experiments in quadruplicate and are expressed as mean ± SD. *p < 0.05.

FCCP is a commonly used protonophore that collapses the mitochondria inner membrane potential disrupting mitochondria function [13]. As shown in Figure 5, a 30-min treatment of MCF-7 cells with 5 and 20 μM FCCP caused a significant increase in MTT reduction, an effect similar to that achieved with Rottlerin at the same times and doses. This result suggests that the Rottlerin interference in the MTT assay is related to its ability to act as a mitochondrial uncoupler.

## 4. Discussion

The MTT assay is a widely used test to measure cell proliferation, cell viability/survival, or drug toxicity. In a recent paper, the use of MTT has been described as one of the major techniques for testing tumor cell resistance to anticancer agents [14]. On the other hand, several papers have reported agents that increase MTT reduction without increasing cell viability [15]. Moreover, some studies reported that certain plant extracts and redox-active polyphenols can interfere with the MTT assay because they directly reduce the tetrazolium salt even in the absence of cells [16-19]. Our results demonstrate that Rottlerin (5 and 20 μM) also strongly enhance the formation of formazan crystals inside cells. However, Rottlerin failed in reducing tetrazolium salts in vitro, indicating that the effect observed in cultured cells is not due to a direct reducing action but that the presence of an intermediate molecule/organelle is needed.

Because the MTT assay is largely based on mithocondrial LDH activity, we hypothesized that Rottlerin could enhance LDH activity indirectly, by maintaining high levels of NADH, due to its H+ donor properties. Rottlerin indeed contains five phenolic hydroxyl groups (Figure 1) that act as hydrogen donors in the scavenging of free radicals, such as 1,1-diphenyl-2-picryl-hydrazyl [9]. The results presented in the current study exclude any Rottlerin modulation of LDH activity in vitro, further confirming the need of whole cells to show the increase in MTT reduction.

Therefore, knowing that Rottlerin can interfere in the respiratory chain by acting as an uncoupler of oxidation and phosphorylation [4], we hypothesized that the Rottlerin artifact in the MTT test could be the consequence of a direct action on mitochondrial respiration. Rottlerin, indeed, by dissipating the inner mitochondrial membrane potential, accelerates electron transfer and increases dehydrogenases activity, oxygen consumption, and NADH oxidation. The observed drop in the cell energy charge indicated that also in MCF-7 cells Rottlerin exerted an uncoupling effect, which accidentally enhanced MTT reduction by over-stimulated mitochondrial dehydrogenases. To verify this hypothesis, a comparative study between Rottlerin and the chemical uncoupler FCCP was performed. The results indicated that the overestimation of the MTT test was linked to Rottlerin uncoupling properties, since both uncouplers, at the same doses, enhanced MTT reduction after only 1-h incubation and roughly to the same extent.

The mitochondrial uncoupling was clearly evident with 20 μM Rottlerin but only weakly apparent with 5 μM Rottlerin. However, since this dose also caused overestimation of the MTT test, it can be hypothesized that even with 5 μM Rottlerin a mild uncoupling occurred in our cells, although could be promptly compensated, in terms of energy metabolism, by an increase in glycolytic ATP production.

In the light of these new findings, we revised, in retrospect, our previous results about the lack of effect of Rottlerin on MCF-7 cell viability [5], since the overestimation of the MTT assay likely masked possible toxic/apoptotic effects in this cell line (false or underestimated result).

Considering that different cells may be not equally sensitive to uncouplers, we also tested the interference of Rottlerin in the MTT assay in another cell type. The results demonstrate that Rottlerin overestimates the MTT assay also in HMVEC, suggesting that such an artifact occurs independently from the cell type.

Our experience indicates that it may not be sufficient to change the medium containing Rottlerin and to wash the cells before adding MTT to avoid a potential bias in concluding results. In fact, the Rottlerin lipophylic nature allows the molecule to freely cross the plasma membrane, to accumulate inside the cell, to target mitochondria, and to exert the well documented uncoupling effects [4]. The Rottlerin intracellular accumulation can be easily verified by a simple observation: The cells remain yellow-colored even after washing.

The results presented in the current paper also indicate that it may not be sufficient to include a control without cells in the MTT assay, a stratagem that has been recommended to avoid false-positive results in the presence of molecules with reducing properties, such as flavonoids [18]. Rottlerin, indeed, does not react with MTT in vitro, but causes enhanced MTT reduction in cultured cells.

Similar to the MTT, the 2,3-bis-(2-methoxy-4-nitro-5-sulfophenyl)-2H-tetrazolium-5-carboxanilide (XTT) measures cell viability based on the activity of mitochondria enzymes in live cells. Unlike the water-insoluble formazan produced from MTT, XTT is readily reduced to a highly water-soluble orange colored product, thus eliminating the need for the solubilization step required for the MTT assay.

Similar to the XTT, the Dojindo's tetrazolium salt, the 2-(2-methoxy-4-nitrophenyl)-3-(4-nitrophenyl)-5-(2,4-disulfophenyl)- 2H tetrazolium, monosodium salt (WST-8), marketed as Cell Counting kit-8 (CCK-8), is reduced by cellular dehydrogenases, in the presence of an electron carrier, to an orange formazan product that is water-soluble.

Despite the technical differences and the detection sensitivity, the MTT, XTT, and WST-8 work on exactly the same principle and measure the same parameter. Therefore, it can be anticipated that the uncoupling properties of Rottlerin also could have a detrimental effect on the results from both XTT and WST-8 cell proliferation assays.

On the basis of our findings, it can be suggested that a safety alternative test in toxicological studies could be the detection of LDH released in the medium by cultured cells, since no interference was observed in our LDH assay in the presence of Rottlerin in the most widely used concentration range (5–20 μM). Some other LDH commercial kits are based on a two steps reaction: (1) the oxidation of lactate to pyruvate and (2) reduction of MTT to formazan by pyruvate. Also these LDH assays can be recommended in the presence of Rottlerin, the drug did not exhibit any direct reactivity toward MTT salts in vitro; thus no interference should be expected.

Moreover, we also discourage the use of the MTT assay in the presence of mitochondrial uncoupling agents; a number of lipophylic molecules, both natural and synthetic, such as polyphenols and steroid hormones, have uncoupling properties. In these cases, the direct cell counting or the direct measurement of cell proliferation (tritiated thymidine or others) seems to be more appropriate than the MTT assay.

Another widely used method to assess proliferation, cytotoxicity, or viability of cultured mammalian cells and to monitor the effects of a wide range of drugs and biological compounds is the measurement of ATP content in lysed cells by colorimetric, fluorometric, and radioisotopic assays. ATP is a good marker for cell viability because it is present in all metabolically active cells, and the concentration declines very rapidly when the cells undergo necrosis or apoptosis. However, although we have not verified a possible interference by Rottlerin and other uncouplers in ATP detection by the methods above, it is possible, or better obvious, that the decrease in ATP caused by mitochondrial uncoupling could be erroneously interpreted as a decrease in cell viability (false or enhanced negative result). We have no suggestions on how to circumvent the interference in these cases and can only discourage the use of ATP measurement in the presence of known or putative uncoupling molecules.

In closing, we have demonstrated in this report that, although the MTT method is the most frequently used in the assessment of the viable cell number, its inadequacy is evident in presence of drugs/compound that cause alterations in mitochondria activity. We observed a significant pitfall in the presence of the chemicals tested, resulting in inaccurate cell number estimation and misinterpretation of results. Therefore, we suggest caution in the use of the MTT assay as a proliferation/viability/toxicity test in the presence of Rottlerin and uncoupling agents in general, especially if not corroborated using alternative/complementary assays.
